# Case report: 18F-FDG PET-CT findings in Bickerstaff encephalitis before and after treatment

**DOI:** 10.3389/fnume.2023.1235173

**Published:** 2023-08-24

**Authors:** Nora E. Kerik-Rotenberg, Jocelyn Cruz-Perez, Ivan E. Diaz-Meneses, Alfredo Javier Aguirre Enriquez, Sarah Elizabeth González Ríos, Emilly A. Cortés-Mancera, Fabio Sinisterra Solís, Francisco Romero Castellanos, Edwin Steven Vargas-Canas, Jesús Ramirez-Bermudez

**Affiliations:** ^1^ Nuclear Medicine Unit, “Manuel Velasco Suarez” National Neurological and Neurosurgical Institute, Mexico City, Mexico; ^2^ Intensive Care Unit, “Manuel Velasco Suarez” National Neurological and Neurosurgical Institute, Mexico City, Mexico; ^3^ Neuromuscular Diseases Clinic, “Manuel Velasco Suarez” National Neurological and Neurosurgical Institute, Mexico City, Mexico; ^4^ Department of Neuropsychiatric, “Manuel Velasco Suarez” National Neurological and Neurosurgical Institute, Mexico City, Mexico

**Keywords:** encephalitis, Bickerstaff, case report, cerebral glucose metabolism, 18F-Fluorodeoxyglucose positron emission tomography

## Introduction

Bickerstaff's Encephalitis (BE) is characterized by a subacute onset of bilateral opthalmoparesis, ataxia, and an altered level of consciousness. Also, pyramidal signs, bilateral facial palsy, bulbar palsy, and pupillary abnormalities are common (1). It falls within the spectrum of anti-GQ1b diseases. Diagnosis uses clinical and laboratory criteria, and imaging techniques allow other diseases to be ruled out. 18F-Fluorodeoxyglucose Positron Emission Tomography (18F]-FDG-PET) in BE has not been extended, so a case with PET follow-up is presented. We present the case of a patient who underwent an [18F]-FDG-PET imaging examination between September 2022 and December 2022 at the Molecular Imaging PET/CT Unit, National Institute of Neurology and Neurosurgery Mexico (Tertiary Neurological Center). In this study, we provide evidence that brain [18F]-FDG-PET may be helpful in identifying likely patterns of regional brain glucose metabolism before and after treatment.

## Case report

A 29-year-old man with a past medical history of chronic headache, hypertension and grade III obesity and with not familiar or psychosocial history, reported 72 h prior to admission, drowsiness, facial asymmetry, blurry vision, dysarthria, progressive muscle weakness and gait disturbance.

On admission at the emergency room, he progressed with profound stupor, bilateral ophthalmoplegia, right peripheral facial palsy, flaccid tetra paresis and generalized hyperreflexia. The Medical Research Council (MRC) Scale for Muscle Strength score was 6 points, which indicates a severe condition. He was intubated and invasive mechanical ventilation was initiated. The diagnostic approach was focused on looking for etiologies of impaired consciousness and muscle weakness.

The complementary tests: blood biometrics (without leukocytosis or leukopenia) and blood chemistry were normal. Toxicological profile was negative. Serology for HIV, hepatitis B-C virus and syphilis were also negative. The cytochemical study of cerebrospinal fluid was within normal parameters and the multiplex Polymerase Chain Reaction (PCR) and culture were negative. The imaging tests, including simple head tomography, CT angiography and head MRI were normal.

Once structural, infectious, vascular, demyelinating, or toxic-metabolic etiologies were ruled out, an autoimmune inflammatory disease was suspected. Given the clinical presentation, Bickerstaff encephalitis was suspected, and electro conduction studies were requested. Severe motor polyradiculopathy, compatible with the AMAN variant (acute motor axonal neuropathy) of Guillain Barré syndrome (GBS), with axonal degeneration affecting all 4 limbs were reported. The determination for antiganglioside antibodies (anti GT1a IgG and anti GQ1b IgG) were positive. As part of the diagnostic approach an [18F]-FDG-PET/CT was ordered.

### Methodology

[18F]-FDG-PET/CT scans were performed using a Biograph 64 mCT PET/ CT system (Siemens Health) in a three-dimensional (3D) mode. CT transmission scan was performed for attenuation correction, as well as scatter and random corrections. Before radiopharmaceutical injection, the patient fasted for at least 6 h. The subject was injected in a dimly lit room without talking or moving. A 10-min PET scan was acquired starting 30 min after the injection of 185 ± 18 MBq of FDG. The visual inspection of all [18F]-FDG-PET scans was done by experienced nuclear medicine physicians (NK, ID, EC, FS, FR). The analysis of the PET images was performed on Siemens Healthcare workstation. Thalamus was used as a reference area for normalization due to its preserved glucose metabolism during this disease. The thalami were easy to delineate using standard semiautomated methods included with the reading software. Other normalization methods such as global mean was not considered because there was a global reduction in cerebral glucose metabolism present in the acute phase of the disease. Similarly, pons as reference area was not used in this case, it was affected during the disease.

**Figure 1:**
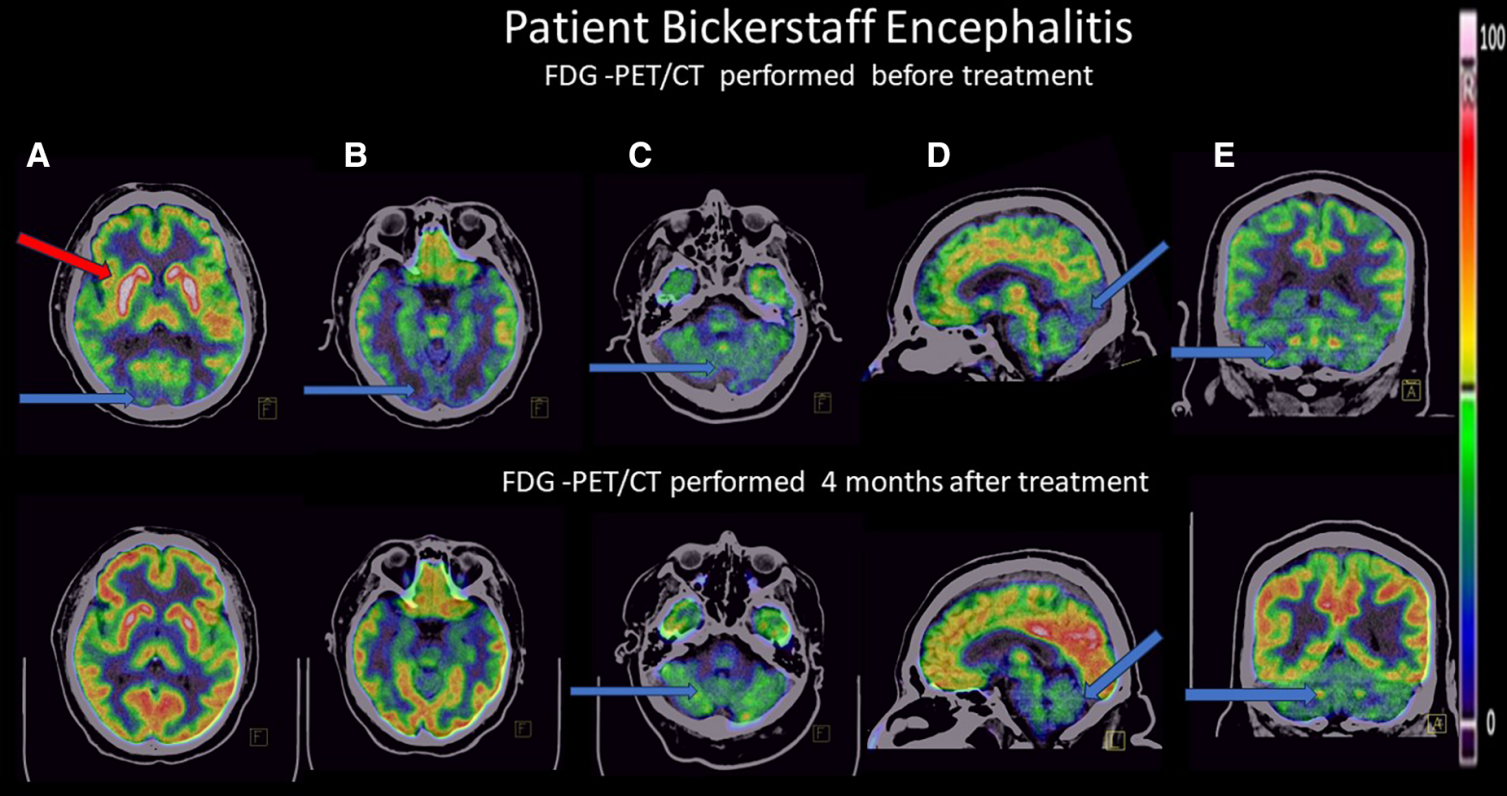
Positron emission tomography (18PET-FDG) brain images from the patient with BBE in 3 orientations (axial, coronal, sagittal views), upper images in the acute phase, lower images 4 months after treatment. Blue arrows show hypometabolism, red arrows show hypermetabolism. Thalamus was used as a reference area for normalization due to its preserved glucose metabolism during this disease. (**A**) Axial view: upper image: mild generalized cortical hypometabolism, severe bilateral striatum hypermetabolism, and severe bilateral occipital hypometabolism; lower image: normalization of FDG metabolism in these areas. (**B**) Axial view: upper image: severe bilateral occipital hypometabolism; lower image: normalization of FDG metabolism. (**C**) Axial view: upper image: severe bilateral cerebellar hypometabolism, lower image: discrete bilateral cerebellar hypometabolism. (**D**) Sagittal view: upper image: mild generalized cortical hypometabolism, severe occipital, cerebellum, brain stem, and vermis hypometabolism bilaterally; lower image: normalization of FDG pattern except for discrete cerebellar, vermis and ventral brain stem hypometabolism. (**E**) Coronal view, upper image: severe occipital and cerebellar hypometabolism bilaterally. Lower image: FDG normalization at the occipital cortex bilaterally, discrete bilateral cerebellar hypometabolism.

PET-FDG findings show bilateral striatum hypermetabolism, mild generalized cortical hypometabolism and severe bilateral occipital, cerebellar, vermis and brainstem hypometabolism (see Figure 1, upper images).

Intravenous immunoglobulin (0.4 g/kg/day) was started for 5 days. His evolution in the Intensive Care Unit was characterized by dysautonomia. He required a tracheostomy to achieve the removal of mechanical ventilation and was discharged with four of 16 points, MRC 50 points. In subsequent controls, the patient managed to reintegrate into his activities of daily living, without alterations of behavior, language, non-involvement of cranial nerves, sensory function, normoreflexia or alterations of gait or cerebellar function. At this moment, the MRC score is 58 points, which is close to the criteria for normality.

**Figure 2:**
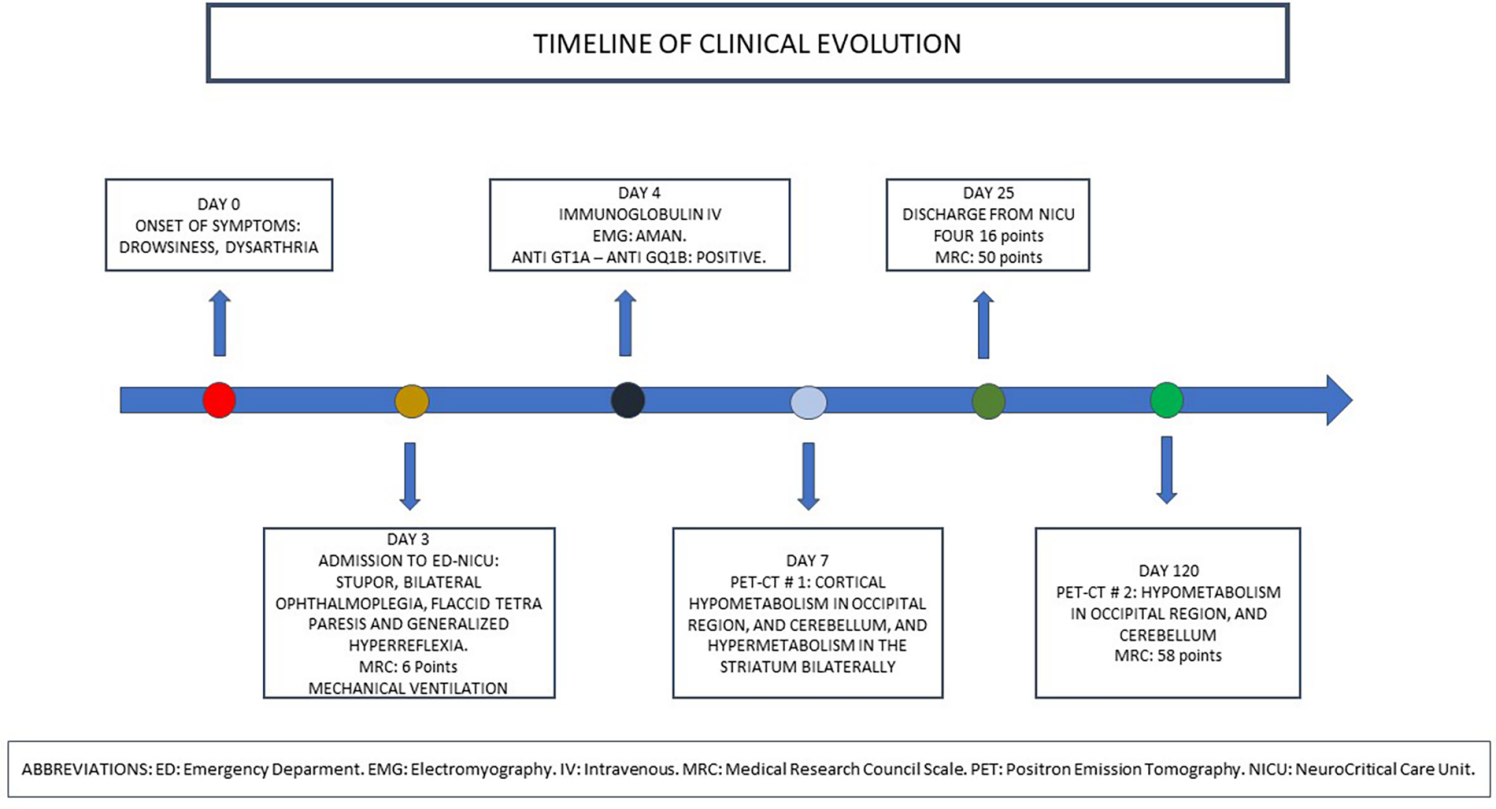
Timeline in the evolution of the patient.

Upon hospital discharge, he achieved functional independence and was in control. After 120 days, he returned to his activities of daily living (see Figure 2).

A second PET-FDG scan was performed 4 months after treatment, with normalization of the FDG pattern except for the persistency of discrete cerebellar, vermis and ventral brainstem hypometabolism (see Figure 1, lower images).

## Discussion

Bickerstaff encephalitis is considered a rare immune-mediated disorder, with an estimated annual incidence of 0.1 per 100,000 in the Japanese population (2). BE It is characterized by ophthalmoplegia, ataxia, and impaired consciousness; a slight predominance in young men has been reported (2, 3). In addition to the clinical picture, the albumin cytological dissociation of the cerebrospinal fluid and the positivity of serum IgG anti-GQ1b antibodies support the diagnosis (4). However, in related entities such as Miller Fisher syndrome and Guillain-Barre syndrome with ophthalmoplegia, positivity for these antibodies has also been reported, even in cases of negativity (5, 6). Regarding our case, the patient fulfilled at onset the Grau's et al. criteria for diagnosis of probable Bickerstaff’s brain stem encephalitis, including 1) rapid progression of less than 4 weeks of decreased level of consciousness, bilateral ophthalmoplegia, and ataxia, as well as the reasonable exclusion of alternative causes. After the positive determination of IgG anti-GQ1b antibodies, the criteria for a definite diagnosis were fulfilled (1).

Given the suspicion of encephalitis, regarding neuroimaging studies as part of the diagnostic approach, MRI and 18F-FDG PET-CT stand out, therefore, it is essential to know their scope and limitations. In the last decade, 18F-FDG PET-CT has been used in the diagnosis of NMDA and other autoimmune brain encephalitis, showing altered patterns of regional metabolism. In the literature, it has been reported that the most consistent metabolic pattern that represents neuronal dysfunction in anti-NMDAR encephalitis is the occipital hypometabolism (7–9).

There is little information other than a few case reports where metabolic alterations have been described in patients with a clinical diagnosis of Bickerstaff encephalitis, such as cerebellar hypometabolism (10), bitemporal and parieto-occipital hypometabolism (11), as well as a report carried out in our institution where findings similar to those described in anti-NMDAR anti-immune encephalitis with hypometabolism in the occipital lobe, cerebellum and striatal nuclei were documented (12).

Although this disease can be difficult to diagnose due to the variety of symptoms, the use of FDG-PET can provide valuable information about metabolic changes in the brain and help in its diagnosis by differentiating it from other neurological diseases with similar symptoms, evaluating the extent and severity of brain inflammation, and monitoring the response to treatment.

We present a case of a patient with a clinical picture suggestive of Bickerstaff encephalitis in which we highlight the alterations in cerebral glucose metabolism using the brain PET technique with FDG and how their metabolic improvement correlated with clinical improvement during follow-up. After immunotherapy, the patients exhibited imaging improvement see Figure 1. 18F-FDG-PET had been reported to show altered patterns of regional metabolism in cases of suspected autoimmune encephalitis, findings observed in our case.

The reversibility of clinical symptoms, and glucose metabolism normalization patterns has usually been linked to reversible receptor function after removing the antibodies.

Our main findings in the baseline 18F-FDG-PET study were mild generalized cortical hypometabolism, severe bilateral occipital, cerebellar, vermis, and brainstem hypometabolism, and bilateral striatum hypermetabolism. Occipital hypometabolism has been observed in patients with severe cognitive abnormalities and a decreased level of consciousness in the context of autoimmune encephalitis (8).

Brain dysfunction changes were not evident at the MR performed on the patient, which gives 18F-FDG PET-CT a high impact as a diagnostic and follow-up method. In a study conducted by Moreno-Ajona. Et al., who evaluated the different metabolic patterns in autoimmune encephalitis, they reported that all patients had significant metabolic changes [with the support of statistical surface projection (SSP) methods], but in 33% of patients who presented metabolic abnormalities, magnetic resonance was normal (13).

### Patient perspective

The patient remarked that the treatment received was optimal in achieving his nearly complete recovery. He emphasizes that he still does not recall what happened during his hospitalization. Furthermore, he comments that it is comforting to know that PET has completely normalized brain metabolism. Finally, he wants to continue with periodic assessments and has made changes in his lifestyle, with the aim of improving his health.

## Conclusion

We report a case of Bickerstaff encephalitis in a patient in the acute phase, and after treatment with intravenous immunoglobulin for five days, it showed redifferentiation and normalization of FDG brain metabolism. It could be an earlier marker for neurological conditions such such as BE, and an excellent tool to aid diagnosis, and provide timely treatment and follow-up. Given the low incidence of BE encephalitis, studies with a larger number of patients are still required, and imaging studies should be performed.

## Data Availability

The original contributions presented in the study are included in the article/Supplementary Material, further inquiries can be directed to the corresponding author.
